# Expression profile of components of the β-catenin destruction complex in oral dysplasia and oral cancer

**DOI:** 10.4317/medoral.24528

**Published:** 2021-09-25

**Authors:** Francisca J Goñi, Daniel Peña‑Oyarzún, Vicente A Torres, Montserrat Reyes

**Affiliations:** 1 Department of Pathology and Oral Medicine, Faculty of Dentistry, Universidad de Chile, Santiago, Chile; 2 Physiology Department, Faculty of Biological Sciences, Pontificia Universidad Católica de Chile, Santiago, Chile; 3 Interdisciplinary Center for Research in Territorial Health of the Aconcagua Valley (CIISTe Aconcagua), School of Medicine, Faculty of Medicine, San Felipe Campus, University of Valparaiso, Chile; 4 Institute for Research in Dental Sciences, Faculty of Dentistry, Universidad de Chile, Santiago, Chile

## Abstract

**Background:**

Oral cancer represents the sixth most common cancer in the world and is associated with 40-50% survival at 5 years. Within oral malignancies, oral squamous cell carcinoma (OSCC) is commonly preceded by potentially malignant lesions, which, according to histopathological criteria, are referred to as oral dysplasia and their diagnosis are associated with higher rates of malignant transformation towards cancer. We recently reported that aberrant activation of the Wnt/β‑catenin pathway is due to overexpression of Wnt ligands in oral dysplasia. However, the expression of other regulators of this pathway, namely components of the β-catenin destruction complex has not been explored in oral dysplasia.

**Material and Methods:**

Using immunohistochemical analyses, we evaluated nuclear expression of β‑catenin and its association with Wnt3a and Wnt5a. Likewise, components of the β-catenin destruction complex, including Adenomatous Polyposis Coli (APC), Axin and Glycogen Synthase Kinase 3 beta (GSK-3β) were also evaluated in oral dysplasia and OSCC biopsies.

**Results:**

We found that moderate and severe dysplasia samples, which harbored increased expression of nuclear β‑catenin, depicted augmented cytoplasmic expression of GSK‑3β, Axin and APC, in comparison with OSCC samples. Also, GSK-3β was found nuclear in mild dysplasia and OSCC samples, when compared with other study samples.

**Conclusions:**

Cytoplasmic levels of components of the β-catenin destruction complex are increased in oral dysplasia and might be responsible of augmented nuclear β‑catenin.

** Key words:**Oral cancer, oral dysplasia, β-Catenin, Wnt ligands, destruction complex.

## Introduction

Oral carcinogenesis is a multifactorial process that involves several molecular and genetic alterations (1). Oral squamous cell carcinoma (OSCC) is the sixth most common malignant cancer of the oral cavity (2). Importantly, only 40-50% of the patients have a 5-year survival, which highlights the relevance of early diagnosis in order to improve patient survival and mortality rates (3).

OSCC is commonly preceded by potentially malignant lesions in the oral mucosa, which are clinically known as leukoplakia and erythroplakia, and are histologically classified as oral dysplasia (2). The diagnosis of dysplasia is usually complex and subjective, since no specific markers are available, and the current knowledge regarding molecular alterations and the evolution of these lesions from early to advanced stages is limited (4).

The Wnt/β-catenin signaling pathway plays an important role in cell growth, differentiation, migration and proliferation (5). β-catenin, the central protein of this pathway, is constitutively sent to proteasomal degradation after being phosphorylated by a cytoplasmic destruction complex formed by the Casein Kinase 1 alpha (CK1α), the Glycogen Synthase Kinase 3 beta (GSK‑3β), Axin and the tumor suppressor protein Adenomatous Polyposis Coli (APC) (6). Binding of extracellular Wnt ligands to Frizzled and LDL receptor-related protein 5/6 (LRP 5/6) receptors triggers the inhibition of β‑catenin degradation, leading to its cytoplasmic accumulation and subsequent translocation to the nucleus, acting as a transcriptional cofactor of genes associated with cell growth and proliferation (6). We and others have demonstrated that nuclear β‑catenin levels are increased in moderate and severe dysplasia, when compared to healthy mucosa and OSCC (7-10), suggesting that accumulation of nuclear β-catenin is an early event during oral carcinogenesis (11).

Aberrant activation of Wnt/β-catenin in cancer has been associated with mutations in components of β-catenin destruction complex, such as APC, Axin and GSK‑3β (12). However, these mutations are not frequent in OSCC, being unlikely that mutations in β-catenin destruction complex may be associated to nuclear β-catenin in oral dysplasia and cancer (11,13,14). The expression and role of Wnt ligands in oral dysplasia is also poorly understood. For instance, Wnt5a is known to promote cell migration and invasion in OSCC (15,16), but modulation of Wnt5a levels during oral dysplasia remains unclear. On the other hand, Wnt3a expression and secretion have been shown to be increased in oral dysplasia samples and *in vitro* cell cultures, respectively (10). Indeed, we recently reported that both nuclear β‑catenin and Wnt3a correlate with advanced dysplasia stages, suggesting that Wnt3a might be relevant for OSCC development (10).

Recent studies from our group showed that endosomal sequestration of the β‑catenin destruction complex is a central event leading to nuclear translocation of β‑catenin in oral dysplasia (11). We demonstrated that upregulation of endosomal proteins, such as Rab5, leads to sequestration of the β-catenin destruction complex and hence nuclear accumulation of β-catenin in oral dysplasia (17). However, the intriguing possibility that components of the destruction complex are deregulated in oral dysplasia, has not been explored yet. Particularly, the association between nuclear β‑catenin and Wnt ligands, as well as components of the β-catenin destruction complex in oral dysplasia, remain unclear. In this study, by using immunohistochemical analysis in oral dysplasia and OSCC biopsies, we evaluated the expression of nuclear β‑catenin and Wnt3a and Wnt5a, as well as components of the β-catenin destruction complex, such as APC, Axin and GSK-3β. We found that moderate and severe oral dysplasia samples depicted increased nuclear β‑catenin levels, which was associated with cytoplasmic expression of GSK-3β, Axin and APC.

## Material and Methods

- Tissue Immunohistochemistry

Case selection. 63 formalin fixed paraffin-embedded samples were collected considering a database from the register of the Pathological Anatomy Service from the Faculty of Dentistry, Universidad de Chile, between the years 2000 and 2019. 30 of these samples had a clinical diagnosis of either oral leukoplakia, oral erythroplakia or oral leukoerythroplakia, as well as a histopathological diagnosis of oral dysplasia. Of these 30 dysplasia samples, 15 samples were classified as mild dysplasia, while the other 15 were classified as either moderate or severe dysplasia. Also, 30 OSCC samples were classified according to their degree of differentiation: 15 cases were classified as well differentiated OSCC, while the other 15 cases were classified as moderately and poorly differentiated. Finally, 3 healthy oral mucosa samples were used as control.

The histopathological diagnosis of each sample included in this study was carried out by two oral pathologists. The demographical and clinical data were directly obtained from the clinical record. This study was approved by the Ethical Committee Board from the Faculty of Dentistry, Universidad de Chile.

Biopsy processing. Sections of 3 μm thickness from paraffin blocks were deparaffinized in xylene and rehydrated in descending alcohols to distilled water. Sections were placed in sodium citrate buffer (pH 6.0) for antigenic recovery and then washed with PBS for 5 min. Endogenous peroxidase activity was blocked by incubating the sections with 3% hydrogen peroxide at room temperature for 10 min. Sections were pre-incubated with horse serum for 30 min at room temperature and incubated with primary antibodies overnight at 4°C. Monoclonal anti-β-catenin (M3539; 1:50 dilution) was from DAKO (Santa Clara, CA, USA), monoclonal antibodies raised against APC (sc-9998; 1:200 dilution), Axin (sc‑293190; 1:100 dilution), Wnt5a (sc-365370; 1:200 dilution) were from Santa Cruz Biotechnology (Santa Cruz, CA, USA). Anti-Wnt3a (ab219412; 1:200 dilution) was from Abcam (Cambridge, UK), while mouse monoclonal anti‑GSK-3β (610201; 1:200 dilution) was from BD Transduction Laboratories (Franklin Lakes, NJ, USA). After washing the sections with PBS for 5 min, they were incubated with biotinylated secondary antibodies for 30 min at 37 °C and with peroxidase-conjugated streptavidin (Universal Detection System Vectastain Elite Kit wide spectrum ABC-HRP, RTU, Vector-USA, EE.UU) for 30 min at 37°C. The reaction was visualized with diaminobenzidine (DAB) and stained with Harris Hematoxylin. Negative controls were obtained by using PBS instead of primary antibodies.

Positive controls were used to determine the ideal concentration for each antibody used in this study. For β-catenin, a colon tissue sample was used; for Wnt3a, a kidney tissue sample was used as positive control; For Wnt5a, a melanoma sample; for APC, a colon tissue sample; for Axin, using a skin sample as positive control; finally, for GSK-3β, an ovary sample was used as positive control.

Sample Analysis. Under microscopic field of 400-fold magnification, the cellular localization of antigens was classified as membranous, cytoplasmic or nuclear, depending on the immunolocalization pattern. Tissues were classified as nuclear positive for β-catenin, if more than 10% of cells showed nuclear staining. Quantification of nuclear immunostaining was performed by analyzing five randomly selected fields per sample, reaching a total of 1000 epithelial cells per sample. The number of cells with positive staining was determined by using the Image J program. Intensity of staining was assessed as mild (+) when the positive staining was achieved within the range of 1-25% of the cells analyzed, while staining was considered as moderate (++) or intense (+++) when positive staining was obtained within the range of 25-75% and 75-100% of the cells analyzed, respectively. In non-homogeneous cases, the predominant intensity (over 75% of cells) was recorded. All immunohistochemical analyzes performed in this study were carried out by two expert observers in the area. The fields were photographed using an Olympus CX41 microscope using the software Micrometrics SE Premium program.

- Statistical analysis

Shapiro-Wilk test was used for an exploratory normality analysis of the data. ANOVA with a Bonferroni post-hoc test was used for statistical comparison between samples. A value of significance of 5% or less (*p* <0.05) was accepted as statistically significant. All statistical tests were performed using Stata 11.0 software.

## Results

- Clinicopathological characterization of the samples studied

**Figure 1:**
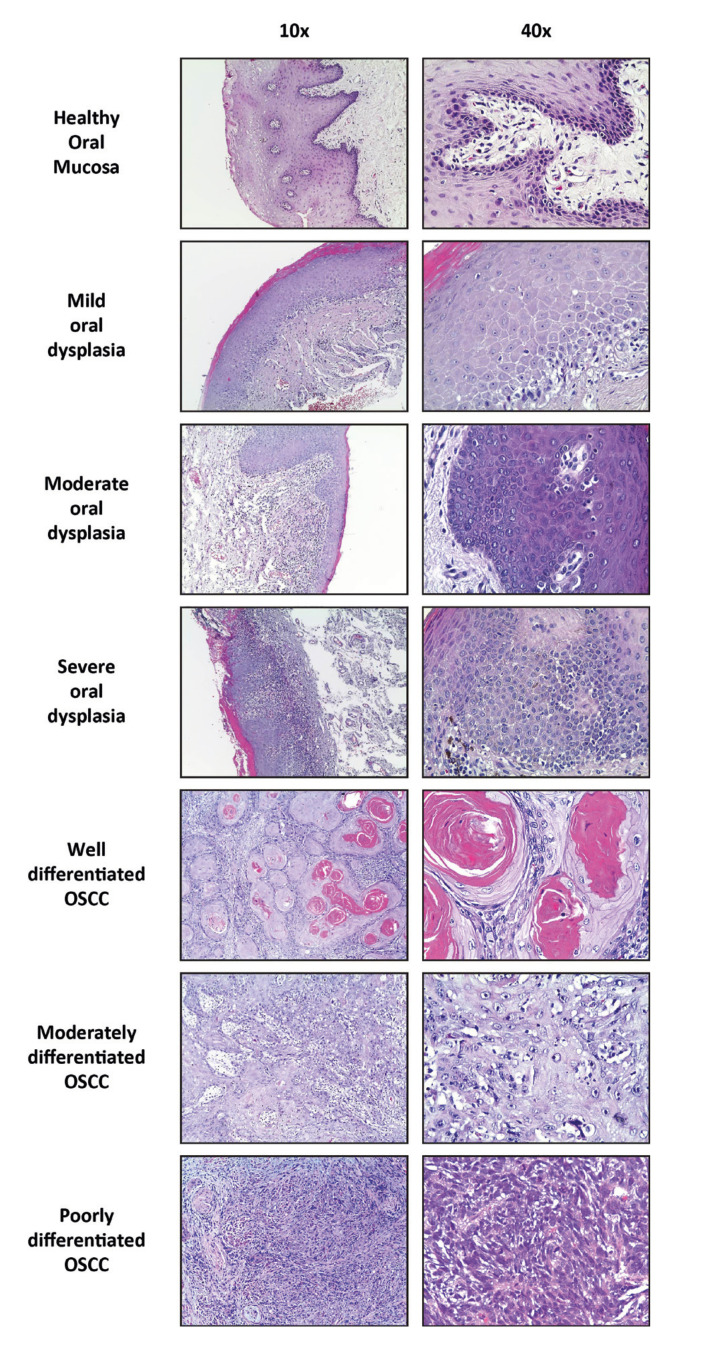
Hematoxylin-eosin staining. Samples of healthy oral mucosa, mild dysplasia, moderate dysplasia, severe dysplasia, well differentiated OSCC, moderate differentiated OSCC, and poorly differentiated OSCC, with hematoxylin-eosin staining.

A total of 63 samples were analyzed: 3 samples of healthy oral mucosa, 30 samples of dysplasia -including 15 samples of mild dysplasia and 15 samples of moderate and severe dysplasia-, and 30 samples of OSCC -corresponding to 15 samples of well differentiated OSCC and 15 samples of moderate and poorly differentiated OSCC- (Fig. 1).

The average age of patients with dysplasia was 60 years, ranging from 51 years to 79 years, while the average age of patients with OSCC was 65 years, ranging from 31 years to 88 years. Mild dysplasia samples were mostly from the tongue, while both moderate and severe dysplasia samples were more frequently obtained from the gum and the mouth floor. OSCC samples were mostly from the tongue and the gum. Table 1 summarizes the clinicopathological features of all cases examined in the present study.

- Increased levels of nuclear β-catenin in severe and moderate oral dysplasia samples

**Figure 2:**
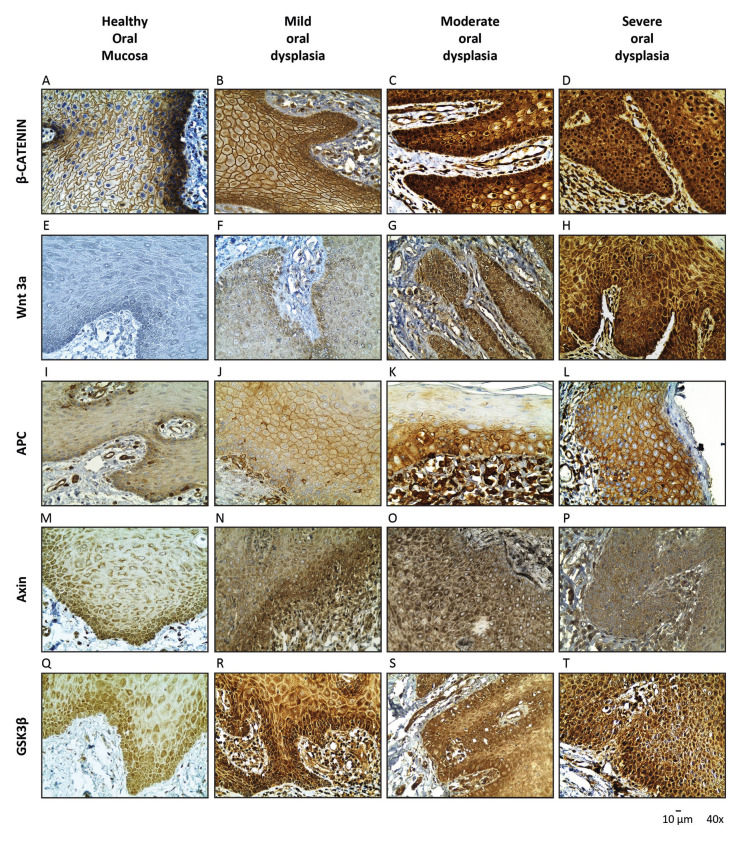
Expression of β-catenin, Wnt 3a, APC, Axin and GSK-3β in Healthy Oral Mucosa and Oral Dysplasia. (A-D) Immunohistochemical levels of β-catenin; (E-H) Wnt 3a; (I-L) APC; (M-P) Axin; and (Q-T) GSK-3β in Healthy Oral Mucosa and mild, moderate, severe oral dysplasia. Representative images are shown. The images were obtained through an Olympus CX 41 microscope, at image capture 40X magnification.

**Figure 3:**
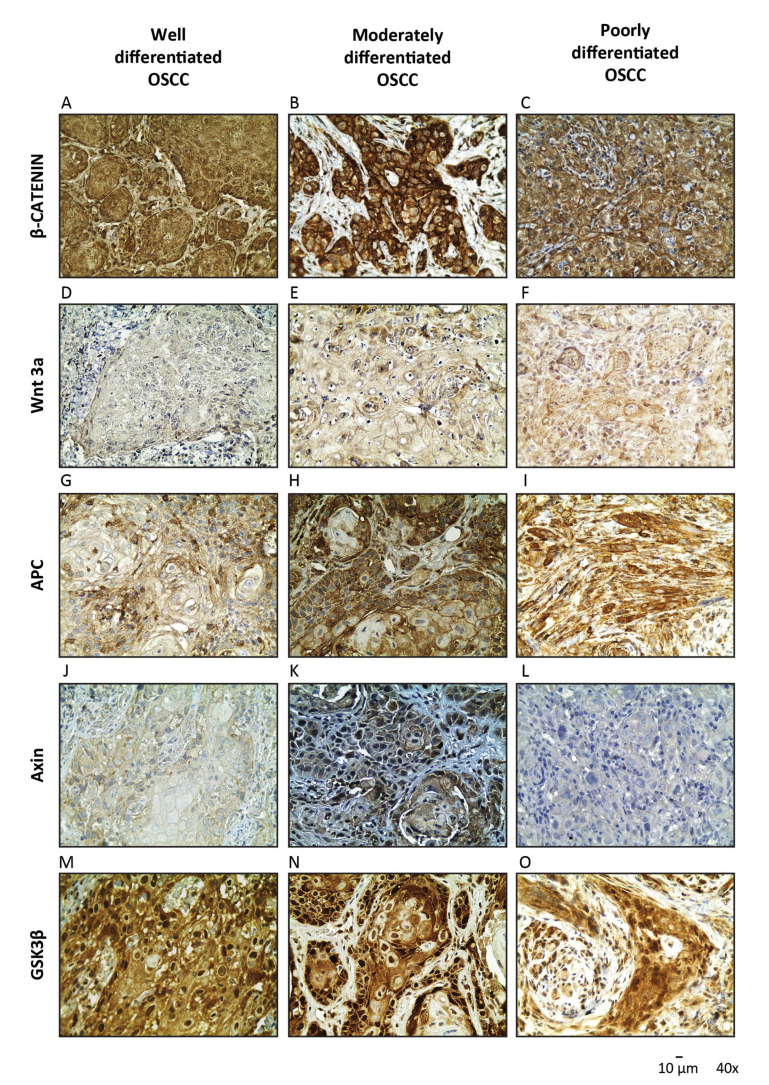
Expression of β-catenin, Wnt 3a, APC, Axin and GSK-3β in OSCC. (A-C) Immunohistochemical levels of β-catenin; (D-F) Wnt 3a; (G-I) APC; (J-L) Axin; and (M-O) GSK-3β in well differentiated, moderately-severe differentiated OSCC. Representative images are shown. The images were obtained through an Olympus CX 41 microscope, at image capture 40X magnification.

**Figure 4:**
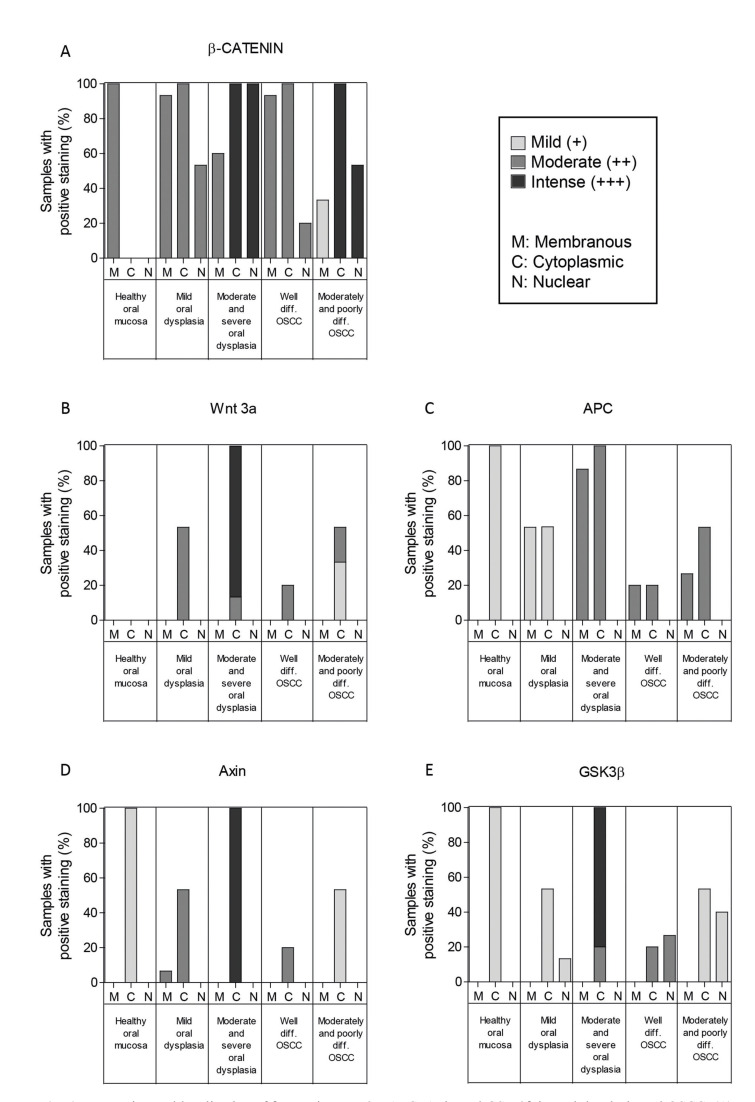
Expression and localization of β-catenin, Wnt 3a, APC, Axin and GSK-3β in oral dysplasia and OSCC. (A) Expression and localization of β-catenin; (B) Expression and localization of Wnt3a; (C) Expression and localization of APC; (D) Expression and localization of Axin; and (E) Expression and localization of GSK-3β in Healthy Oral Mucosa, mild, moderate, severe oral dysplasia and well differentiated, moderately-severe differentiated OSCC.

In all healthy oral mucosa samples, localization of β-catenin was detected at the cell membrane, with negative immunostaining at the cytoplasm and the nucleus (Fig. 2). Nuclear expression of β-catenin in epithelial dysplasia was found to be increased and associated to the grade of dysplasia (Fig. 2). The tissues with moderately and severe dysplasia showed a 100% of nuclear positivity, in contrast to mild dysplasia tissues, were a 53% of nuclear positivity was observed. Significant differences were determined when comparing mild dysplasia and healthy oral mucosa samples with severe oral dysplasia samples (*p* < 0.05). Similarly, severe oral dysplasia samples showed predominant nuclear localization of β‑catenin when compared with OSCC samples, where a cytoplasmic location of the antigen was frequently observed (*p* < 0.01) (Fig. 3). These results suggest that nuclear β-catenin levels increase during advanced stages of dysplasia, validating the previous observations made by our group (8,10). The expression and localization of β‑catenin in the samples are summarized in Fig. 4.

**Table 1:**
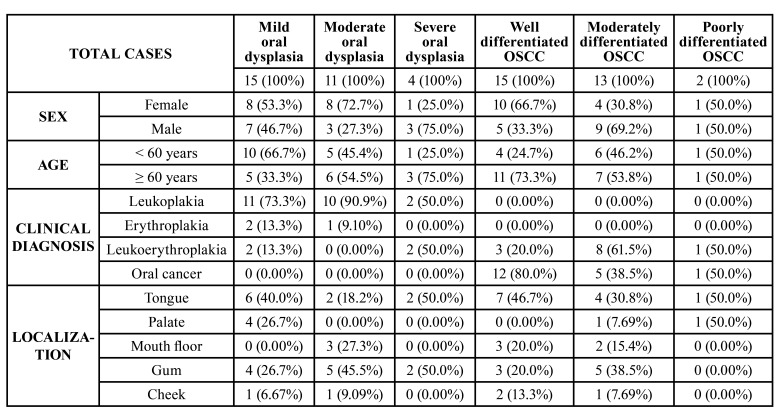
Clinicopathological and demographical features of the samples. Biopsies were diagnosed as mild, moderate or severe oral dysplasia, while OSCC biopsies were diagnosed as well differentiated, moderately differentiated, or poorly differentiated.

- Increased expression of Wnt3a in samples of severe oral dysplasia

 Of all the dysplasia and OSCC samples analyzed (n=60), 100% of epithelial cells expressed Wnt3a in all epithelium strata but, unexpectedly, it was not expressed in the healthy oral mucosa (n=3) (Fig. 2). Interestingly, epithelial cytoplasmic expression of Wnt3a correlated with nuclear expression of β-catenin in oral dysplastic samples (Fig. 2). Oral moderate and severe dysplasia samples showed higher intensity of Wnt3a when compared to healthy oral mucosa and mild dysplasia samples (*p* <0.01) (Fig. 2). As well, oral moderate and severe dysplasia samples displayed higher intensity than all OSCC samples, regardless of the OSCC grading (*p* <0.01) (Fig. 3). Expression and localization of Wnt3a are summarized in Fig. 4.

The expression of Wnt5a also was analyzed, however no differences were found between the study groups (data not shown). This suggests that that Wnt3a, but not Wnt5a, is required for Wnt signaling activation at advanced dysplasia stages. These results support previous observations where secretion of Wnt3a is required for the activation of the Wnt/β‑catenin and the expression of genes in oral dysplasia (10).

- Increased expression of APC and Axin in severe oral dysplasia samples

 In the healthy oral mucosa samples, APC was located at the cytoplasmic level (Fig. 2). However, localization of APC in dysplasia samples was mostly membranous. Interestingly, in 86.6% of the severe dysplasia samples the membranous localization of APC correlated with a nuclear expression of β-catenin (Fig. 2).

Also, moderate and severe dysplasia samples showed higher intensity of APC than mild dysplasia and healthy oral mucosa samples (*p* <0.05).No differences were found in APC intensity in other study groups. Expression and localization of APC are summarized in Fig. 4.

We also checked the levels of Axin, another component of the β-catenin destruction complex. All oral dysplasia and healthy oral mucosa samples depicted cytoplasmic location of Axin (Fig. 2). In moderate and severe oral dysplasia samples, a moderate expression of Axin was correlated with nuclear expression of β‑catenin (Fig. 2). Although the OSSC samples also presented cytoplasmic location of Axin, its intensity was progressively decreasing when moving from a higher to a lesser degree of differentiation (*p* <0.05) (Fig. 3). Expression and localization of Axin are summarized in Fig. 4.

- Increased levels of nuclear GSK-3β in OSCC samples compared to oral dysplasia samples

All oral dysplasia samples showed cytoplasmic GSK-3β localization. Interestingly, two mild dysplasia samples showed both cytoplasmic and nuclear localization of GSK-3β (12% of nuclear positivity), as well as absence of nuclear expression of β-catenin (Fig. 2). In moderate and severe dysplasia samples, a higher cytoplasmic intensity was observed when compared to mild dysplasia and healthy oral mucosa samples (*p* <0.05).

Like in oral dysplasia samples, OSCC samples also displayed a cytoplasmic localization of GSK-3β. Curiously, 4 well-differentiated OSCC samples and 6 moderate/poorly differentiated OSCC samples showed both cytoplasmic and nuclear expression of GSK-3β, as well as absence of nuclear expression of β-catenin. The samples of well-differentiated OSCC showed a 20% of nuclear positivity, while in moderate/poorly differentiated OSCC a 40% of nuclear positivity for GSK-3β was observed (Fig. 3). Expression and localization of GSK-3β are summarized in Fig. 4.

## Discussion

Aberrant activation of Wnt/β-catenin is commonly associated with malignant progression in several cancers (5). We have previously demonstrated that nuclear localization of β-catenin is increased in oral dysplasia samples (8) and that this increase is associated with augmented secretion of Wnt3a *in vitro* and ex vivo models (10). However, to the date, the role of proteins belonging to the β-catenin destruction complex, has not been fully elucidated in oral dysplasia. We addressed this previously, using an *in vitro* approach to propose a mechanism where the endosomal sequestration of the β-catenin destruction complex may be key for nuclear β-catenin translocation in oral dysplastic cells (17). Despite this, no study has documented the expression levels of the β-catenin destruction complex proteins in patient’s biopsies, nor its association to Wnt ligands and β-catenin expression, during the progression from oral dysplasia to OSCC.

It is known that increased expression of nuclear β-catenin is associated with moderate and severe oral dysplasia, while decreased membrane expression of β-catenin is associated with worse prognosis of OSCC (8,18,19). In accordance with previous immunohistochemical studies from our group (8,10) and others (7,9), the results showed here indicate that nuclear β‑catenin expression is increased in moderate and severe dysplasia, compared to mild dysplasia and OSCC samples.

Aberrant activation of the β-catenin pathway has been associated to increased secretion of Wnt ligands, leading to augmented translocation of β‑catenin to the nucleus in different types of cancer (10,20-22). Among the Wnt ligands, Wnt3a and Wnt5a are the most studied regarding oral epithelial cell proliferation and differentiation (7,23-25). Indeed, our results show that Wnt3a levels are considerably increased during dysplasia and OSCC. However, no differences are observed for Wnt5a, indicating that the contribution of Wnt signaling to the progression of dysplasia to OSCC is rather specific for Wnt3a (10) and this work). Although aberrant activation of Wnt signaling pathway has been mainly associated with mutations in the components of the β‑catenin destruction complex (26,27), mutations in these components are not frequent in oral carcinogenesis (13,14). The latter suggests that overexpression of Wnt3a leads to the activation of Wnt signaling, leading to the sequestration of APC, Axin and GSK-3β (10,17). Indeed, in this study we show that the intensity of APC is significantly higher in moderate and severe dysplasia, compared to mild dysplasia and healthy oral samples. As well, we show that Axin intensity was higher in oral dysplasia, compared to OSCC samples. Previous studies demonstrated that APC and Axin play an important role in nuclear export of β-catenin (28), but there are no studies linking the role of these proteins with oral carcinogenesis. Here, we demonstrated that augmented expression of these proteins in oral dysplasia is associated with nuclear accumulation of β‑catenin, which could indicate a carcinogenic role for APC and Axin.

It is known that the activation of Wnt signaling through ligand-receptor binding inhibits GSK-3β‑mediated phosphorylation of β-catenin, leading to cytoplasmic accumulation of β-catenin and translocation to the nucleus (6). Some studies indicate that an increase of cytoplasmic expression of GSK-3β could be associated with OSCC (17), but no studies analyzed its expression in oral dysplasia. Our findings show that moderate and poorly differentiated OSCC samples depict increased nuclear localization of GSK-3β, compared to oral dysplasia samples, which show mostly a cytoplasmic localization. GSK-3β also controls nuclear β-catenin function (5,6). Indeed, some studies suggest that nuclear localization of GSK-3β inhibits the function of nuclear β‑catenin, acting as a transcriptional cofactor that promotes tumorigenesis independently of β‑catenin (29). Accordingly, Caspi *et al*., reported in colon cancer cell lines that nuclear GSK-3β decreased nuclear β‑catenin activity (29). Thus, our study would be the first one to report a correlation between nuclear GSK-3β and β-catenin in oral carcinogenesis, where nuclear GSK-3β could be associated to the loss of nuclear β-catenin expression in OSCC, probably decreasing β-catenin/TCF-dependent transcription.

In summary, we propose that nuclear detection of β-catenin in oral dysplasia is associated to the activation of Wnt signaling pathway by the Wnt3a ligand. This leads to nuclear accumulation of β-catenin and cytoplasmic increase of the components of the β-catenin destruction complex in dysplasia. However in OSCC, nuclear GSK-3β may behave as a negative regulator of nuclear β-catenin, promoting tumorigenesis by Wnt-independent routes.
